# On the Microscopic Anatomy, Physiology and Pathology of the Human Liver

**Published:** 1864-05

**Authors:** H. D. Schmidt

**Affiliations:** Surgeon P. A. C. S.


### CONFEDERATE STATES MEDICAL & SURGICAL JOURNAL.

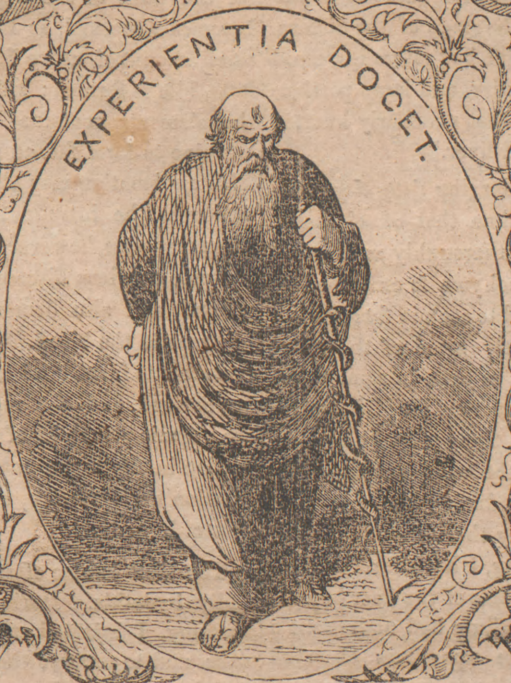

VOL. I. RICHMOND, MAY, 1864. No. 5.

### ORIGINAL COMMUNICATIONS.

ART. 1.—*On the Microscopic Anatomy, Physiology and Pathology of the Human Liver.* By H. D. SCHMIDT, Surgeon P. A. C. S. (Concluded.)

Having now obtained a correct idea of the intricate arrangement of the anatomical elements, that compose the human liver, we are better enabled to build a sound pathology upon it. In regard to the *organic* diseases to which this organ is subject, I have little to say. Want of time and suitable opportunity have prevented me from investigating them as thoroughly as I am in the habit of doing. Nevertheless, it shall not interfere with the statement of the views I have formed from the observations I have made. The consideration of the malignant diseases, however, as carcinoma, &c., I have to defer to some other time, for the subject is too extensive to be treated in this place. It will be ample material for a monograph. Therefore, my remarks will be confined to the liver in a state called cirrhosis, and to that of fatty degeneration. In regard to the former I have had several opportunities to make hasty examinations of specimens in that condition, and had even commenced, several years ago, to make preparations for a thorough investigation of this interesting subject. But circumstances interfered with my intentions, and there the matter rested. However, from what I have seen and read on the subject, I am inclined to think that *cirrhosis* consists in an hypertrophy of the capsule of the "portal vessels," perhaps also that of the "hepatic veins." These capsules, in becoming hypertrophied, and extending over all the vessels of the organ, of course would encroach upon the parenchyma, or proper secreting substance, and the latter necessarily become atrophied. But, independent of this mechanical pressure exerted upon the parenchyma, the blood vessels being also compressed, and having their calibre reduced, are unable to further supply the organ with an amount of blood sufficient for its nourishment and secretion. Thus, while the fibrous tissue of the capsule becomes more developed and more dense, the blood-vessels and parenchyma become more compressed, and consequently diminished in size and quantity. The organ not being able any longer to remove those principles, of which the bile is composed, from the blood, the secretion becomes diminished, elements that ought to be eliminated from the blood become obnoxious to the system by remaining there, and as the disease progresses it at last ends in death. *Frerichs,* in his work on diseases of the liver, speaks of the presence of spindle-formed, and, if I remember rightly, also stellated cells in cirrhosed livers. I have looked for these cells, while examining some specimens, but could not find them. In tearing up a small portion of parenchyma, however, there are often found fragments of the meshes of capillaries, which have the appearance of spindle-formed or stellated cells, and might easily be mistaken for them. When the liver becomes cirrhosed, it very frequently becomes diminished in size and harder, according to the stage of the disease. Nevertheless, I have seen specimens, which had rather increased in size, although the substance was harder, and large portions of the surface of the organ presented that peculiarly knotted appearance, commonly called "hob-nail liver."

The condition of the liver when underging *fatty degeneration,* I have more closely examined than that of cirrhosis, though, for the want of time, not as thoroughly as I would desire. When a portion of the parenchyma is examined in this state, the hepatic cells will be found to contain fat globules, varying in number and size; frequently some of the latter run together and form very large ones, so that, in some cases, they almost fill up the whole cell. Numerous fat globules are also found free, *i. e.*, between the cells, these most likely were at first formed and contained within the cells, but were discharged by the bursting of the latter during the process of secretion, and refused to be absorbed by the biliary tubules or by the lymphatics. In several instances, I have carefully dissected, under low magnifying powers, some of the finer hepatic ducts, and found that even their epithelium had undergone a fatty degeneration. Fat globules are sometimes observed within hepatic cells of normal human livers, but the number of these instances is not sufficient to warrant the idea that the fat is a natural secretion, and I therefore regard it as a deviation from health.

The human liver, in a state of fatty degeneration, is often called the "Drunkard's Liver." It is true, that persons who are in the habit of drinking freely of strong alcoholic liquors, often become the subjects of this disease; but the disease is also found in persons of opposite habits. One of the finest specimens of "fatty liver" I ever met with, I found by making a *post-mortem* examination of a child (between three and four years old) that died with Bright's disease, or fatty degeneration of the kidneys. The latter disease I had diagnosticated by the examination of the urine, but the former I never suspected. The cause of fatty degeneration of the liver is not the same in all cases. With the drunkard it is said that the disease is caused by an excess of carbon, taken into the system in the form of alcohol. The lungs being unable to eliminate all the carbon, the liver is called upon to assist. But as the vital forces with which it is endowed suffice only for the secretion of a certain quantity of bile, without destroying the integrity of the organ, much of the carbon remains unconsumed. For the want of vitality now to transform this carbon along with other elements into bile, it is left to combine with hydrogen to form an organic principle of a lower order, namely, fat. Another explanation might be as follows: the lungs not being able to eliminate the excess of carbon, the liver readily responds to the call to assist in the task. But in doing so, it is obliged to draw very largely for an additional amount of nerve force from the nerve centres by which it is supplied. The latter, by the constant drain, and not being able to generate more than a certain amount, at last become depressed and exhausted, and in this condition cannot even furnish the quantity essential to the process of a normal secretion. Now, for the want of the necessary nerve stimulus, the secretion becomes abnormal, as in any other organ. As long as the small amount of nerve force, by which the liver is still supplied lasts, bile is formed; but as soon as the vitality is exhausted, the elements, contained in a state of excess in the plasma, combine to form fat, an organic principle of a lower order.—This explanation may also be applied to those cases not originating in the introduction of alcohol into the system. In these instances, however, the nervous system becomes exhausted by some previous disease, reducing the tone of the system in general, and also decreasing the normal amount of vitality inherent to the blood. The latter, being deficient in vitality, is unable to furnish a healthy plasma from which all secretions are derived, and the result is that some of the productions are organic principles of a lower order, like fat. Fatty degeneration in any organ may always be traced back to a state of inaction or depression, either of the organ concerned or of the system in general.

The liver is also often the seat of parasitic animals, especially hydatids. During my researches into its microscopic anatomy, I injected and examined the livers of many animals, and to procure those of the sheep, hog, &c., uninjured, for that purpose, I usually removed them from the animals myself at the slaughter-house. While thus engaged, I found, to my astonishment, that a very considerable number of livers of the hog were infested with hydatids; sometimes of great size, as large, perhaps, as the fist. On inquiring into the cause, the butchers told me that the hogs, whose livers were thus diseased, had been fattened mainly on corn.

The *functional* diseases of the liver are those in which no additional products are formed, and no change of structure, either in quality or quantity, takes place within it. But the most of them are accompanied by a singular phenomenon, called *icterus* or *jaundice;* and it is to the explanation of this that I wish to direct the attention of the medical profession. It is hardly necessary, in this place, to dwell long on the theories that have heretofore existed, and still exist, in explanation of jaundice. They can be found in most treatises on the practice of medicine. The one I was formerly taught was that the liver, being in a torpid condition, failed to abstract from the blood the constituents of which the bile is composed. The coloring matters of the latter would thus accumulate in the blood, and produce the yellow appearance of the skin, by which they at last, if the torpidity of the liver persisted, would be excreted; if the skin could not remove them all, the kidneys would come to the rescue. But before proceeding to the true explanation of the phenomenon, I will first present a fact which has been noted by many observers. This is, that in organic diseases of the liver, where the secreting powers of the liver are evidently diminished by an encroachment on, and a consequent diminution of its parenchyma, jaundice is a very rare occurrence; but that, on the contrary, when jaundice is followed by death, the parenchyma is found to be unaltered, but the hepatic ducts are very generally in a diseased condition. *Lehmann*, in his excellent treatise on organic chemistry, directs our attention to these facts, while treating on the subject of bile. I will accept, then, the fact, that in jaundice the hepatic ducts are very generally diseased, and use it as a foundation for my arguments in the explanation of that phenomenon. In the meantime, however, I must refer to the microscopic anatomy of the liver, as I have described it in these pages, and especially to one of the triple communications that exists between the lymphatics and the hepatic ducts. It will be remembered that the finest branches of the lymphatics take their origin in the same capillary net-work of "biliary tubules," in which the finest hepatic ducts commence. Supposing now, that, by some cause or other, the mucous membrane of the hepatic ducts becomes inflamed, and in consequence of this inflammation swells and becomes thickened; of course, by the thickening of the mucous membrane, the calibre of this duct or tube must evidently become diminished, or even obliterated. But, in the meantime, the parenchyma or secreting structure of the liver, is in a perfectly normal condition, and the secretion is going on as usual. Now, I ask, what must be the natural consequence of this condition of things? In casting a glance at the diagrams, the answer is ready. The circulation of the blood is going on without interruption; the constituents of which the bile is to be composed are carried to the parenchyma as usual, and within the hepatic cells they are elaborated into bile. When the process of secretion is completed within the cells, they burst, and discharge their secretion into the space they occupied before bursting. The secretion is absorbed by the net-work of "biliary tubules," and thence enters into the hepatic ducts. It is now that arises the difficulty, when the secretion has arrived at the place of partial, or even entire obstruction, caused by the thickening of the mucous membrane of the hepatic ducts. And what is the result of this obstruction? Why, the secretion going on as usual, and being unable to find its natural outlet through the hepatic duct into the duodenum, must evidently accumulate behind the obstruction, or seek another outlet. At first, the smaller hepatic ducts become overfilled, and next the net-work of "biliary tubules." But as the secretion is still going on, and the obstruction is not removed, the bile seeks another outlet, which it soon finds in the lymphatics that arise from the net-work of "biliary tubules"—Hence, it is an easy matter to perceive what is to follow. The obstruction in the hepatic duct still existing, the bile now accumulates in the finer lymphatics, from these it gets into the larger lymphatic vessels, and at last arrives in the thoracic duct. The latter, in its turn, discharges it into the left subclavian vein, and thus finally it arrives in the cavity of the heart. Here it becomes thoroughly mixed with the blood, and then enters the general circulation. The presence of bile in the blood is first detected in the sclerotic coat of the eye, the whiteness of this coat, and the transparency of the conjuction, affording a greater contrast to the yellowness of the bile than the skin. But the bile, as a finished secretion, and especially its coloring matters, being excrementitious, is now a foreign substance to the blood; and as it might become injurious to the whole system, it must be eliminated. I do not pretend to say that the liquid portion of the bile is necessarily injurious or fatal to the system; it may be only the coloring matters; for the former, as already mentioned, is re-absorbed in the small intestines. The skin at first performs the vicarious function of the elimination of the bile or its coloring matters through its numerous perspiratory glands; and in such cases, both the skin and perspiration are intensely yellow, the latter often staining a white handkerchief when wiped off. If the action of the skin is insufficient for the elimination, the kidneys assist, and the bile can be detected in the urine by chemical tests. Besides the glands of the skin and kidneys, other gland and secreting membranes may participate in the elimination, particularly the salivary glands and the mucous membrane of the mouth, as evinced frequently by the presence of a bitter taste in cases in which the functions of the liver are deranged. The mucous membranes of the air-passages may even assume a vicarious function of the liver, as illustrated by a case related to me by my friend, Surgeon D. W. Brickell.* However, in many cases of jaundice, notwithstanding the elimination of bile by the perspiratory glands of the skin, yellowness of the skin persists for a considerable time, although the liver has resumed its normal functions, and the patient entirely recovered from the functional derangement of this organ. In these instances, the yellow appearance of the skin is due to the presence of bile within the cells of the epidermis, thrown out from the capillaries of the skin along with the plasma, from which those cells were formed. The jaundice, of course, will gradually disappear as the epidermical cells are thrown off and replaced by others formed from a plasma containing no bile.

In the preceding lines, I have merely given a sketch of the rationale of jaundice in general. Various circumstances, of course, will modify or arrest the phenomenon in its course.—These I shall now consider:

In some cases, the obstruction, arresting the flow of bile, and caused by a swelling of the mucous membrane, lining the bile duct, may exist to such an extent as to only diminish

---

* The following is Dr. Brickell's own statement: In November, 1862, I was called to attend Mr. H., at the Confederate House, Jackson, Mississippi. He was under the care of my friend, Dr. S. Choppin, who was obliged to leave for Charleston. Mr. H. had arrived several days before from Richmond. He had long been afflicted with diarrhœa, and on his arrival from Richmond, was thoroughly jaundiced. I was called to treat him for a severe attack of pneumonia. When I first saw him, he was expectorating mucus of a decidedly yellow tinge; this tinge became rapidly more intense, and in a day or two the handkerchiefs into which he expectorated would become thoroughly saturated, and dyed as yellow as saffron. This feauture was so striking as to attract the attention of all who entered the room. The coloring matter was evidently coughed up from the lungs, as I have frequently seen the patient in the act of expectoration.—D. W. B.

the calibre of this canal without obliterating it. In such a case, a certain amount of bile would still arrive in the duodenum, and the functions of the intestines not be materially deranged. Nevertheless, the obstruction may be sufficient to cause a regurgitation of bile, and the entrance of the latter into the lymphatics and the blood; a yellowness of the eyes will then be observed, and the skin also may assume a yellow tinge; but as soon as the obstruction is removed, the latter disappears. Therefore, the intensity of the jaundice, and the time of its duration, depend on the extent of the degree and duration of the obstruction. In other cases, only the hepatic ducts of a portion of the liver may be affected, while the rest, and also the large hepatic duct, remain untouched by disease, and consequently open to afford an outlet to the bile secreted in the healthy portion of the organ. Now, if the affection is only partial and transient, the system at large may not be materially disturbed; but when the obstruction exists in the common hepatic duct, and extends so as to obliterate its calibre, the consequences will be of a more serious nature. In warm climates, in the spring of the year, the weather sometimes becomes suddenly very hot and oppressive. The atmosphere, becoming more heated, expands, and is rarified, in consequence of which the supply of oxygen, essential to the lungs to perform their function in the elimination of carbon from the system, is diminished. The carbon, accumulating within the blood, seeks another outlet, which it finds through the liver; but the latter, in order to eliminate this carbon, must increase the amount of its secretion; and as, in a normal condition of things, only a certain amount of bile can pass through the hepatic ducts to enter the duodenum, the surplus must accumulate behind, and, as already explained, enter the lymphatics, the result of which will be the phenomenon of jaundice. A number of such cases have come under my observation and treatment in New Orleans in the spring of 1861, especially in the month of May. In these cases, an abnormal amount of bile secreted, is poured into the duodenum, and, in many instances, enters the stomach, producing great distress and obstinate vomiting of bile. When this occurs, it is very remarkable, how the presence of an excessive amount of bile in the duodenum should reverse the peristaltic action of the muscular fibres of this part of the intestines, directing the flow of this secretion towards the stomach; and that also the circular muscular fibres, surrounding the pyloric orifice of the latter, should relax to allow it to enter into its cavity by passing the pyloric valve. Such cases are usually accompanied by obstinate constipation, often very difficult to remove. However, in other instances, the reversion of the peristaltic action of the muscular fibres does not take place, and the bile is allowed to take its natural course through the intestinal canal; but being present in an abnormal quantity, it over-stimulates the muscular fibres, and also irritates the mucous membrane. The irritation of the latter produces at first diarrhœa, but, if persistent, may terminate in inflammation, aggravate the diarrhœa, and even extend through the canal, and result in dysentery.

I have seen many cases of jaundice, and especially among our soldiers, accompanied by a very slight, if any, disturbance of the system in general. The patient feels almost as well as ever, and the functions of the system are apparently little or not deranged, though in many cases the yellowness of the eye and skin persist for a considerable time. This variety of jaundice has been designated "Camp Jaundice" by many surgeons of the army. Though the pathology of it is undoubtedly the same in all cases, *i. e.*, a congestion or a slight inflammation of the mucous membrane of the common hepatic duct or some of its branches, the affection may be created by different causes. One of the latter may be the influence of cold, producing the condition of said membrane just mentioned, similar to that of the Schneiderian membrane in the affection called "coryza." This membrane, which lines the cavity of the nose, is very susceptible to an impression of cold, made either directly upon it, or upon the general surface, and reflected to it. My own person has furnished me a favorable subject of observation. Being very susceptible to the influence of cold, I have often observed that when sitting quietly in a cold room engaged in reading or writing, or when being exposed for a short time to a direct current of air, I suddenly commence to sneeze, and before long, if the impression of cold has been continued long enough, the passages of my nose will be almost closed by the congestion of the lining mucous membrane, so that I have to breathe mainly through the mouth. Avoiding the cause, however, the membrane soon recovers its integrity, (sometimes in one night,) and the affection disappears. Now, the same may take place in the mucous membrane lining the hepatic duct and its branches. The soldier, having been on the march during the day, throws himself upon the cold and damp ground, when arriving in his camp at night. The mucous membrane of the bile ducts becomes congested, the calibre of the canal is diminished, and jaundice appears, accompanied by no other symptoms of disease. If the cause is avoided, the congestion of the membrane will depart, the bile in the blood will be eliminated by the skin, and the jaundice disappears. Another cause may be of a miasmatic nature. The miasma, reaching the blood through the medium of the lungs, circulates through the body. When arrived at the nerve centres, it makes its first impression. The extent of the impression depends on the intensity of the cause; if the latter exists in a slight degree, it may only embrace the centres which supply one or the other organ with nerve force. In the case under discussion, the nerve centres, supplying the lining membrane of the bile ducts, become affected, and they fail in generating the amount of nerve force, essential to the preservation of the tone of the membrane; the arterioles, venules and capillaries of the latter become relaxed, and a congestion ensues. A most remarkable case, in which jaundice suddenly appeared by a strong mental emotion, was not long since related to me by a medical gentleman. It corroborates my explanation of the production of this phenomenon by an affection of the nerve centres. In the same manner, jaundice is produced, when accompanying intermittent and bilious remittent fever, modified, of course, by circumstances. The two latter diseases, together with

"pernicious or congestive fever," being produced by the same cause, I regard as one disease, differing only in degree. Their pathology is, as I have already pointed out, namely, the miasma leaves its first impression on the nerve centres; through the latter the respectives organs they supply become affected. The nervous phenomena, presented to us in these diseases, are so manifold and interesting that I cannot properly discuss them in this place; they will at some other time afford me ample material for a special treatise. The many cases that came under my observation and treatment in the year 1861 at New Orleans, both in the wards that I held in the Charity Hospital and in my private practice, afforded me a fine opportunity for making a special study of the pathology and treatment of these diseases. Having taken notes of the most interesting cases for illustration, and also made some microscopic observations, regarding the nature of miasma, I commenced to write an extensive treatise on the subject; the manuscript, however, as far as it went, I left behind me at New Orleans when entering the army. Nevertheless, as soon as practicable, I shall make it the subject of a monograph.

When the nerve stimulus, essential to any secreting organ, to perform regularly its normal functions, becomes perverted, the productions also become perverted or abnormal; and this is frequently the case in the liver, when the bile becomes altered in its composition. While engaged in my researches into the microscopic anatomy of the human liver, I, of course, had ample opportunity to examine the bile found in the gall-bladders of the numerous specimens I used. Though these specimens, as far as they themselves were concerned, were healthy in their structure, yet they were livers of subjects that had come to death by various causes. Some were taken from men who were hurled out of life by some violent accident, others again from patients who died from the effects of an acute or chronic disease of some other organ. In these specimens, then, I found that the bile, taken from the different gall-bladders, differed very much in color and density. The shades of color ranged from the bright yellow through the brown and green, up to the black; and the density from that of milk to that of a viscid fluid, like tar. In one specimen, taken from a woman who died of typhoid fever, I especially recollect that the bile, contained in the gall-bladder, was as black and viscid as tar; in others, especially that taken from a young man who died of chronic bronchitis, accompanied by disease of the kidneys, it was as bright as gold. In many diseases of other organs, the functions of the liver become disturbed in various degrees, and, as a natural consequence, the secretion must deviate from the normal standard. But, independent of other organs, and even in apparent health, the liver is very liable to have its functions slightly deranged, when the bile will become altered in its composition.

During the spring and summer seasons of warm climates, by the long continued heat of the weather, the system frequently becomes depressed; and the blood-vessels, having lost their healthy tone, are relaxed, in consequence of which the circulation of the blood is carried on in a sluggish and languid manner. Usually the first effect of this relaxation is felt in the liver. This organ, notwithstanding its participation with the other organs in the general relaxation of the system, and with scarcely sufficient ability to perform properly its own functions, is called on to assist the lungs in the elimination of carbon. In some cases it may succeed in performing the additional function, but in others, a congestion of its blood-vessels is the result. When the blood-vessels of the liver become congested, and the congestion is not removed, it must at last extend to the veins of the other abdominal viscera, which contribute to the formation of the portal vein. Such a congestion, if persistent, gives rise to various complaints, as hemorrhoids, pain in the bowels, stomach, &c., and will even keep up an existing diarrhœa or dysentery. The pain in the stomach or bowels is not acute, but, on the contrary, dull and heavy, resembling very closely the pain experienced in dyspepsia. A number of these cases has come under my observation, and I have cured them by relieving the congestion in the portal circulation.

Much might I say yet in regard to the various functional derangements of the liver, and the symptoms to which they give rise, if I had more time at my disposal; but as the object I had in view, *i.e.*, the explanation of the phenomenon of jaundice, is accomplished, I shall herewith close my remarks on the pathology of this organ.

